# Prostaglandin F_2α_ (PGF_2α_) production possibility and its receptors expression in the early- and late-cleaved preimplantation bovine embryos

**DOI:** 10.1186/s12917-019-1939-0

**Published:** 2019-06-14

**Authors:** Katarzyna Grycmacher, Dorota Boruszewska, Emilia Sinderewicz, Ilona Kowalczyk-Zięba, Joanna Staszkiewicz-Chodor, Izabela Woclawek-Potocka

**Affiliations:** Department of Gamete and Embryo Biology, Institute of Animal Reproduction and Food Research, Polish Academy of Sciences, 10-747 Olsztyn, Poland

**Keywords:** Embryo, Bovine, Prostaglandin F_2α_

## Abstract

**Background:**

Prostaglandin F_2α_ (PGF_2α_) is an important component for the physiology of female reproductive processes. In the literature, the data pertaining to the synthesis and action of PGF_2α_ in early embryonic bovine development are limited. In our study, we used the bovine in vitro culture model based on the time of first cleavage to determine the mRNA expression and immunolocalization of PGF_2α_ synthase and its receptor in bovine embryos from the 2-cell stage to the hatched blastocyst stage. We also evaluated PGF_2α_ production at 2, 5 and 7 days of in vitro culture.

**Results:**

We found a significantly higher proportion of blastocysts obtained from the early-cleaved embryos than from the late-cleaved embryos (37.7% vs. 26.1% respectively, *P* < 0.05). The PGFS mRNA expression was significantly higher in the late-cleaved group than in the early-cleaved group at the 2-, 4- and 16-cell stages (P < 0.05). For PTGFR, we observed that within the late-cleaved group, the mRNA abundance was significantly higher in embryos at the 2- and 16-cell stages than in embryos at the 4- and 8-cell stages (*P* < 0.05). We observed that PTGFR mRNA expression was significantly higher in the 2- and 16-cell embryos in the late-cleaved group than that in the early-cleaved group embryos (*P* < 0.05). Among the blastocysts, the PGFS and PTGFR expression levels showed a trend towards higher mRNA expression in the late-cleaved group than in the early-cleaved group. Analysis of PGF_**2α**_ production showed that within the early-cleaved group, the content of PGF_**2α**_ in the in vitro culture medium was significantly higher on day 7 than it was on day 2 (*P* < 0.05).

**Conclusions:**

The mRNA expression levels of PGF_2α_ synthase and its receptor depend on the developmental stage and the embryo quality. Analyses of PGFS and PTGFR expression in bovine blastocysts and of PGF_2α_ embryo production suggest that prostaglandin F_2α_ can act in an autocrine and paracrine manner in bovine in vitro-produced preimplantation embryos. Moreover, the tendency of PTGFR and PGFS mRNA expression to be upregulated in embryos with low developmental potential can indicate a compensation mechanism related to high PGFS and PTGFR mRNA expression levels in low-quality embryos.

## Background

Prostaglandin F_2_ alpha (PGF_2α_) and other prostanoids are synthesized from arachidonic acid (AA), which is converted into unstable prostaglandin H_2_ (PGH_2_) by either prostaglandin endoperoxide synthase 1 or 2 (PTGS1 or PTGS2) [1, 2]; PGH_2_ is then metabolized into PGF_2α_ by the 9,11-endoperoxide reductase (referred to as prostaglandin F_2α_ synthase (PGFS); originating from the aldo-keto-reductase 1C (AKR1C) family) or through aldose reductase with 20α-hydroxysteroid dehydrogenase activity (AKR1B5, originating from the AKR1B5 family) [2–4]. The alternative pathway of PGF_2α_ biosynthesis involves the reduction of PGE_2_ by 9-keto-prostaglandin-reductase (9 K-PGR), which is localized in the endometrium, placenta and corpus luteum of ruminants [5–8]. PGF_2α_ exerts its biological action through prostaglandin F_2α_ receptor (PTGFR), which belongs to the group of G-protein-coupled receptors. Prostanoids belong to the group of biologically active lipids, which are well-known primary mediators of pathological conditions such as inflammation and cancer but are also essential for the physiology of female reproductive processes [9]. In cows, increased uterine concentrations of PGF_2α_ are correlated with high embryonic mortality and decreased pregnancy rates [10, 11]. Moreover, the addition of PGF_2α_ to culture medium decreases the in vitro development of rabbit [12] and rat [13] embryos as well as the in vitro and in vivo development of bovine embryos [14]. Additionally, Hockett et al. [15] showed that the administration of PGF_2α_ exerts a negative influence on embryo quality and the ability to develop past the morula stage.

The developmental competence of oocytes is closely related to their quality and is the key limiting factor in the effective performance of assisted reproduction techniques in females. During in vitro embryo production, the selection of competent oocytes is crucial for successful fertilization, correct embryo development and subsequent high pregnancy rates. The knowledge of oocyte and embryo physiology during certain, defined steps of in vitro development may stimulate the further development of other techniques, such as the marker-assisted and genomic selection of preimplantation embryos or even benefit-assisted reproduction in human beings. There are several crucial steps during in vitro bovine embryo development. After fertilization, the zygote undergoes cleavage divisions into the 2-, 4-, 8- and 16-cell stages. Following this, at 32–64 cells, the blastomere overcomes polarization, which results in the compaction and formation of the morula stage. Compacting embryos encompass cellular events, including the development of Ca^2+^-dependent cell adhesion, the formation of gap junctions for cell communication between blastomeres, the initiation of cell contact-induced polarization, and the formation of cell-to-cell tight junctions, which can divide the plasma membranes of the outer blastomeres into apical and basolateral membrane domains. Thereafter, the complex process of cavitation results in blastocyst formation. During cavitation, trophoectoderm development occurs, which initiates the first contact with the uterus during implantation [16].

In the literature, we can find two types of models for the investigation of oocyte developmental competence. Lonergan et al. [17] and Patel et al. [18] described the bovine model based on the time of the first cleavage. According to their studies, good quality embryos cleave early, approximately 30 h post-fertilization, whereas embryos with low developmental competence cleave 6 h later. It has also been documented that early-cleaved bovine embryos develop to the blastocyst stage at a higher rate than their late-cleaved counterparts [17]. The differences in the timing of the first cleavage result in a dissimilarity in transcriptome composition between the early- and late-cleaved embryos at the two-cell stage [18]. However, the difference in the rigorous, time-dependent expression of the defined gene programme between the early- and late-cleaved embryos at all the previously mentioned crucial stages of their preimplantation development remains unclear. This time-dependent expression of the defined gene programme remains to be an area of particular interest for our research and includes the determination of the expression of enzymes, receptors and factors involved in PGF_2α_ synthesis and action. Taking the above into consideration in our studies, we planned to describe the specific pathway of PGF_2α_ synthesis and its receptor expression in early- and late-cleaved preimplantation bovine embryos during the crucial stages of their development in vitro. Moreover, we examined PGF_2α_ production by early- and late-cleaved bovine embryos on days 2, 5 and 7 of in vitro culture.

## Results

### Developmental rates of the early- and late-cleavage embryos at day 5 and day 7 of in vitro culture

**Fig. 1:**
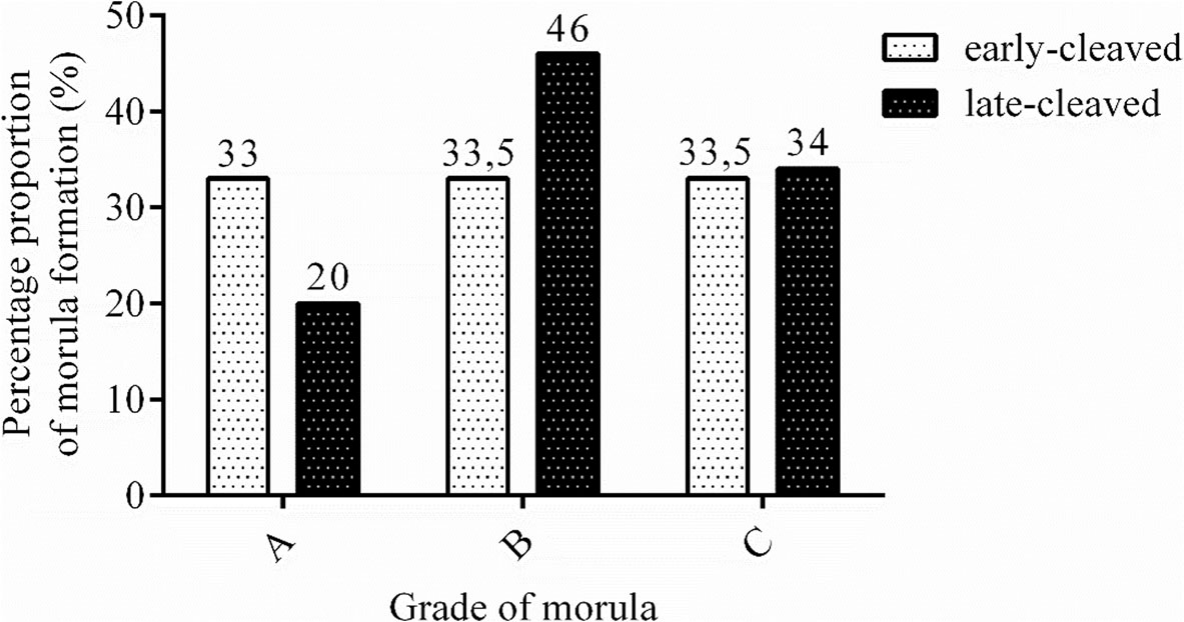
Distribution of morula quality in early- and late-cleaved groups. The figure demonstrates the proportion of morulas at grades A, B and C in the early- (white bars) and late-cleaved groups (black bars). The values are presented as percentages of embryos within each group. The numbers above the bars show the precise percentage of the embryos from each category

**Fig. 2:**
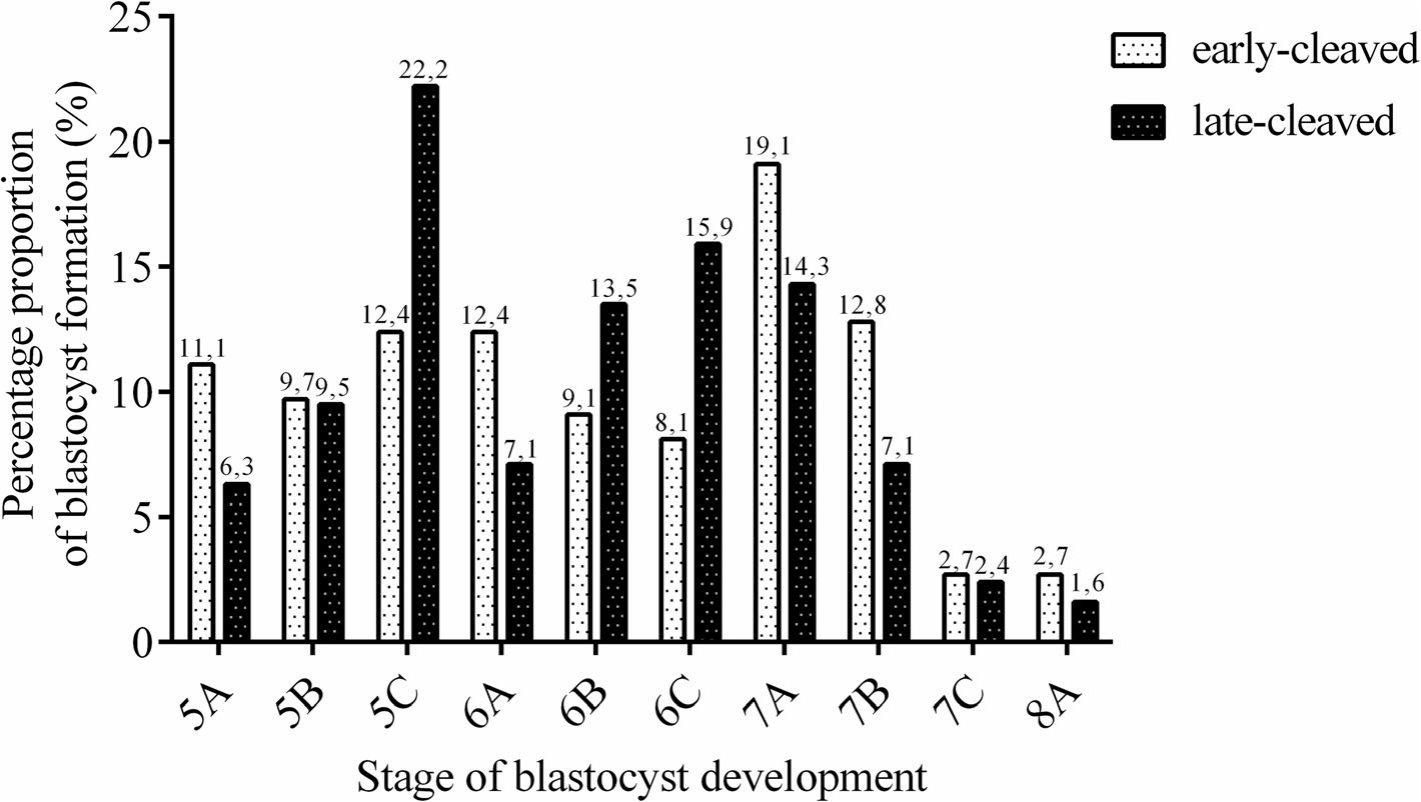
Distribution of blastocyst quality in early- and late-cleaved groups. The figure demonstrates the proportion of early blastocysts (5), blastocysts (6), and expanded blastocysts (7) at grades A, B and C and the grade A hatched blastocysts (8) in the early- (white bars) and late-cleaved groups (black bars). The values are presented as the percentages of the embryos within each group. The numbers above the bars show the precise percentage of the embryos from each category

Table 1 presents the developmental rates of the morulas from the early- and late-cleaved groups on day 5 of in vitro production (IVP). We did not find any differences in morula formation between the analysed groups (58% vs. 62.5%, *P* > 0.05, Table 1). We found a significantly different quality contribution from the two groups of examined embryos based on the phase of the first cleavage (32.8% vs. 39.7%, *P* < 0.05, Table 2). Moreover, on day 7 of IVP, we detected a significantly higher proportion of blastocysts obtained from the early-cleaved embryos than from the late-cleaved embryos (37.7% vs. 26.1%, respectively, P < 0.05, Table 2). Figures 1 and 2 present the contributions of the different qualities and stages of development of the obtained morulas and blastocysts within the early- and late-cleaved embryos. We observed that among the early-cleaved group of morulas, there was a similar percentage of embryos in each grade, but in the late-cleaved morula group, we noticed that the amount of grade A morulas was lower than the amount of grade B and C morulas. Within the blastocysts, we detected that in the early-cleaved group, the most numerous was the 7A group, but in the late-cleaved group, 5C was the most numerous. Furthermore, we noticed that among the late-cleaved group of blastocysts, the number of blastocysts increased from grade A to C for the early and developed blastocysts but decreased from grade A to C in the expanded group of blastocysts. For the early-cleaved group of embryos, we detected a slow decline from grade A to C in the developed stage of blastocysts and a much more pronounced reduction in the number of expanded blastocysts.

**Table 1:** Morula formation rates depending on the time of first cleavage

number of fertilized oocytes, n	fertilization rate, n (%)	early-cleaved embryos (30 hpi), n (%)	late-cleaved embryos (48 hpi), n (%)	morula formation rate, n (%)	morulas from early-cleaved embryos at Day 5, n (%)	morulas from late-cleaved embryos at Day 5, n (%)
1242	794	336	458	420	166	254
	(63)	(27)^a^	(36)^b^	(52,9)	(58)	(62,5)

Proportion of embryos cleaved at 30 and 48 h post insemination (hpi) and formation of morulas at Day 5 of in vitro culture depending on the time of the first cleavage. Percent of early- and late-cleaved embryos was presented according to the number of fertilized oocytes. Morula formation rate was calculated according to the total number of cleaved embryos. Percent of early- and late- cleaved morulas was presented in relation to the total number of embryos cleaved at 30 and 48 hpi, respectively

^a,b^ Differences between cleavage rates (*P* < 0.0001)

*P* values of cleavage rate determined by Chi-square test with Yates’ correction

**Table 2:** Blastocysts formation rates depending on the time of first cleavage

number of fertilized oocytes, n	fertilization rate, n (%)	early-cleaved embryos (30 hpi), n (%)	late-cleaved embryos (48 hpi), n (%)	blastocyst formation rate, n (%)	blastocysts from early-cleaved embryos at Day 7, n (%)	blastocysts from late-cleaved embryos at Day 7, n (%)
5794	4202	1900	2302	1313	716	600
	(72,5)	(32,8)^a^	(39,7)^b^	(31,3)	(37,7)^A^	(26,1)^B^

Proportion of embryos cleaved at 30 and 48 h post insemination (hpi) and formation of blastocysts at Day 7 of in vitro culture depending on the time of the first cleavage. Percent of early- and late-cleaved embryos was presented according to the number of fertilized oocytes. Blastocyst formation rate was calculated according to the total number of cleaved embryos. Percent of early- and late- cleaved blastocysts was presented in relation to the total number of embryos cleaved at 30 and 48 hpi, respectively

^a,b^ Differences between cleavage rates (*P* < 0.0001)

^A,B^ Differences in blastocysts formation rates between early and late cleaved group (*P* < 0.0001)

*P* values of cleavage and blastocysts rate determined by Chi-square test with Yates’ correction

### The expression profile of PGFS and PTGFR in the preimplantation embryos

**Fig. 3:**
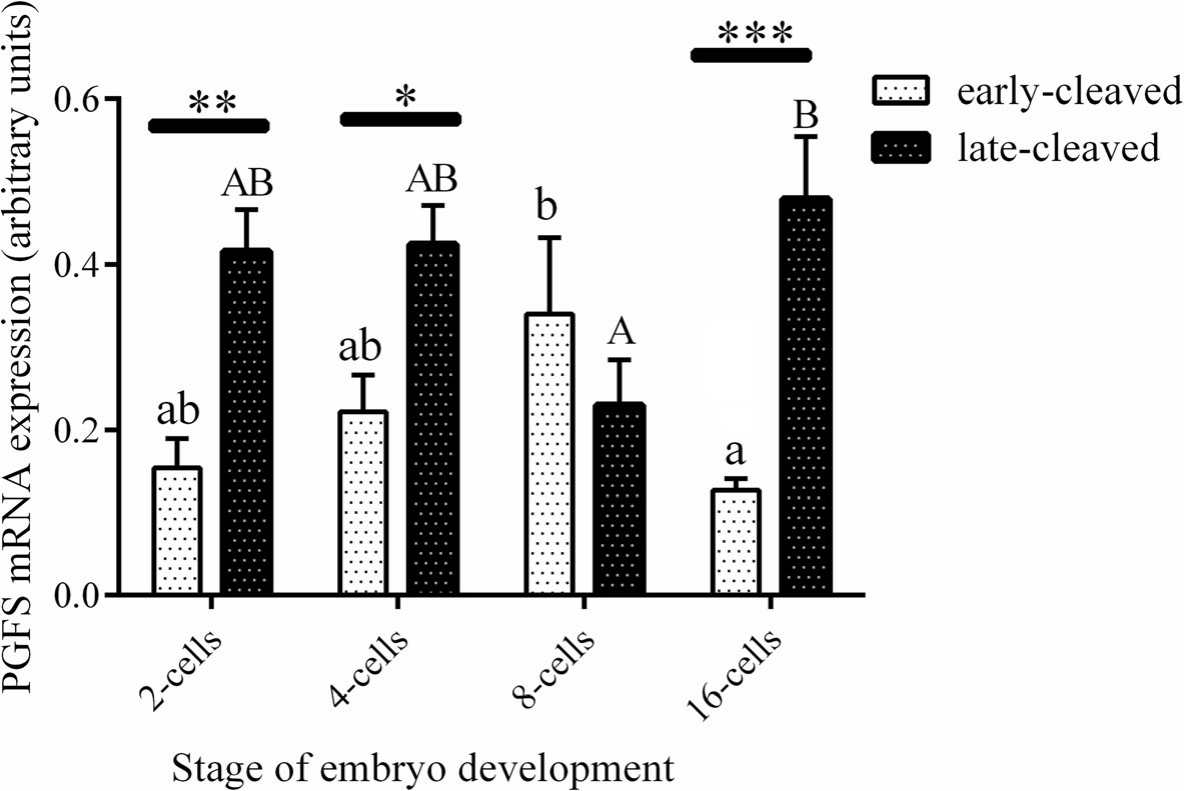
The transcription profile of PGFS in bovine early- and late-cleaved embryos at the 2- to 16-cell stages. The values are presented as arbitrary units and are expressed as the mean ± SEM. Different small letters indicate statistical significance (*P* < 0.05) within the early-cleaved group (white bars), different capital letters indicate significant differences (*P* < 0.05) within the late-cleaved group (black bars), and asterisks indicate statistical significance (*P* < 0.05) between the early- and late-cleaved groups, as determined by two-way ANOVA, followed by the Bonferroni’s multiple comparison test

**Fig. 4:**
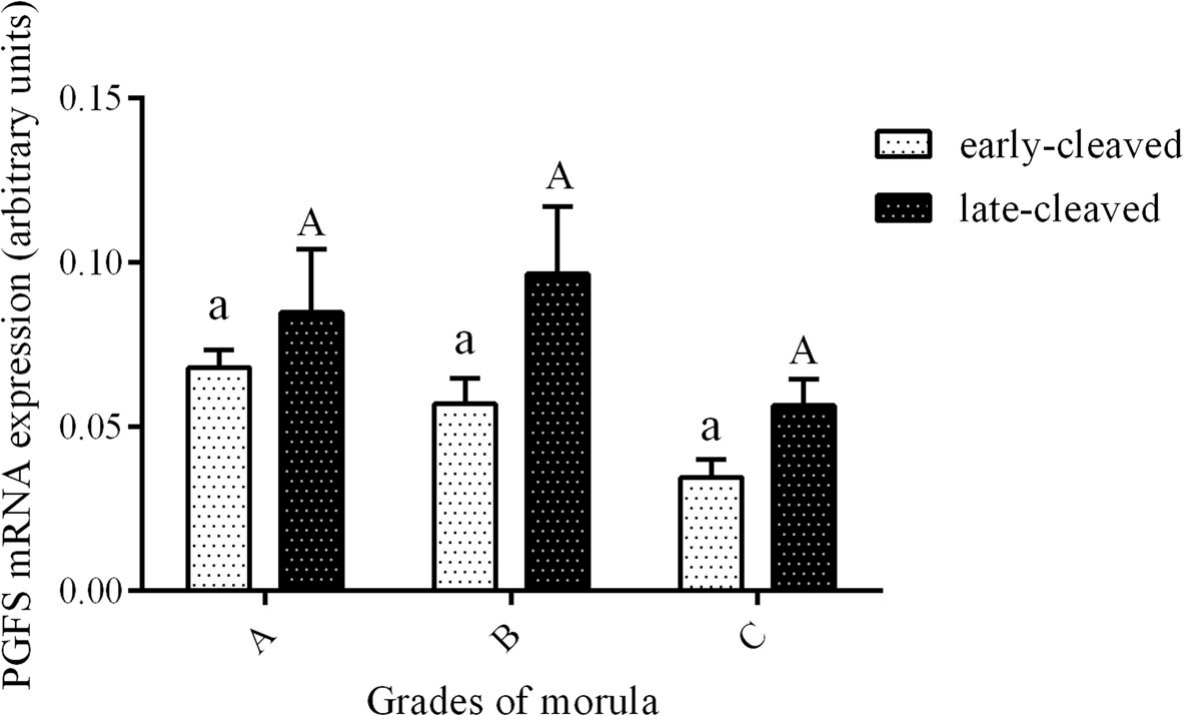
The transcription profile of PGFS in bovine early- and late-cleaved embryos at the morula stage. The figure demonstrated the quality grade-dependent changes in mRNA expression in morulas at grades A, B and C within each group. The values are presented as arbitrary units and are expressed as the mean ± SEM. There were no significant differences (*P* > 0.05) within the early- (white bars) and late-cleaved groups (black bars) or between particular grades of morula stage (*P* > 0.05), as determined by two-way ANOVA, followed by the Bonferroni’s multiple comparison test

**Fig. 5:**
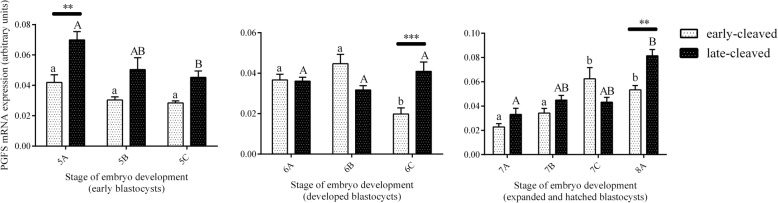
The transcription profile of PGFS in bovine early- and late-cleaved embryos at the blastocyst stage. The figure demonstrated the changes in mRNA expression in (A) early blastocysts (5), (B) blastocysts (6) at grades A, B and C, and (C) expanded blastocysts (7) at grades A, B and C and grade A hatched blastocysts (8) within each group. The values are presented as arbitrary units and are expressed as the mean ± SEM. Different small letters indicate statistical significance (*P* < 0.05) within the early-cleaved group (white bars), different capital letters indicate significant differences (*P* < 0.05) within the late-cleaved group (black bars), and asterisks indicate statistical significance (*P* < 0.05) between the early- and late-cleaved groups, as determined by two-way ANOVA, followed by the Bonferroni’s multiple comparison test

**Fig. 6:**
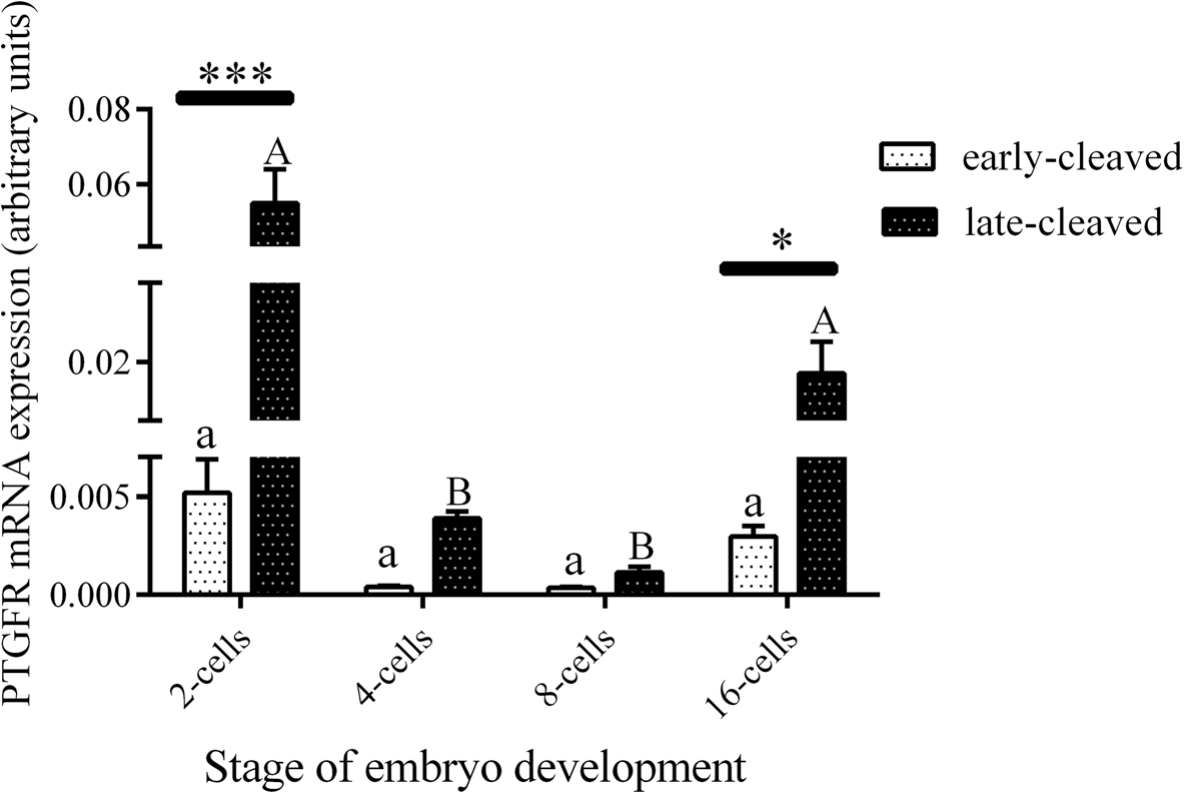
The transcription profile of PTGFR in bovine early- and late-cleaved embryos at the 2- to 16-cell stages. The values are presented as arbitrary units and are expressed as the mean ± SEM. Different small letters indicate statistical significance (*P* < 0.05) within the early-cleaved group (white bars), different capital letters indicate significant differences (*P* < 0.05) within the late-cleaved group (black bars), and asterisks indicate statistical significance (*P* < 0.05) between the early- and late-cleaved groups, as determined by two-way ANOVA, followed by the Bonferroni’s multiple comparison test

**Fig. 7:**
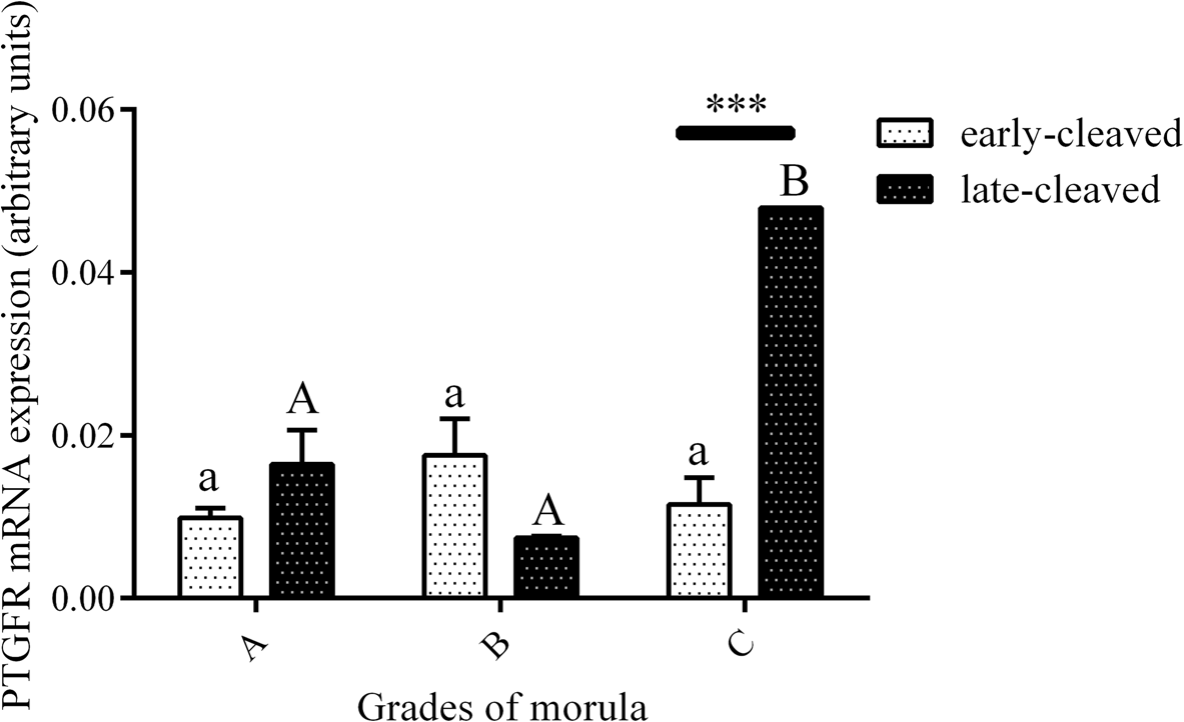
The transcription profile of PTGFR in bovine early- and late-cleaved embryos at the morula stage. This figure demonstrates the quality grade-dependent changes in mRNA expression in morulas at grades A, B and C within each group. The values are presented as arbitrary units and are expressed as the mean ± SEM. Different small letters indicate statistical significance (*P* < 0.05) within the early-cleaved group (white bars), different capital letters indicate significant differences (P < 0.05) within the late-cleaved group (grey bars), and asterisks indicate statistical significance (P < 0.05) between the early- and late-cleaved groups, as determined by two-way ANOVA, followed by the Bonferroni’s multiple comparison test

**Fig. 8:**
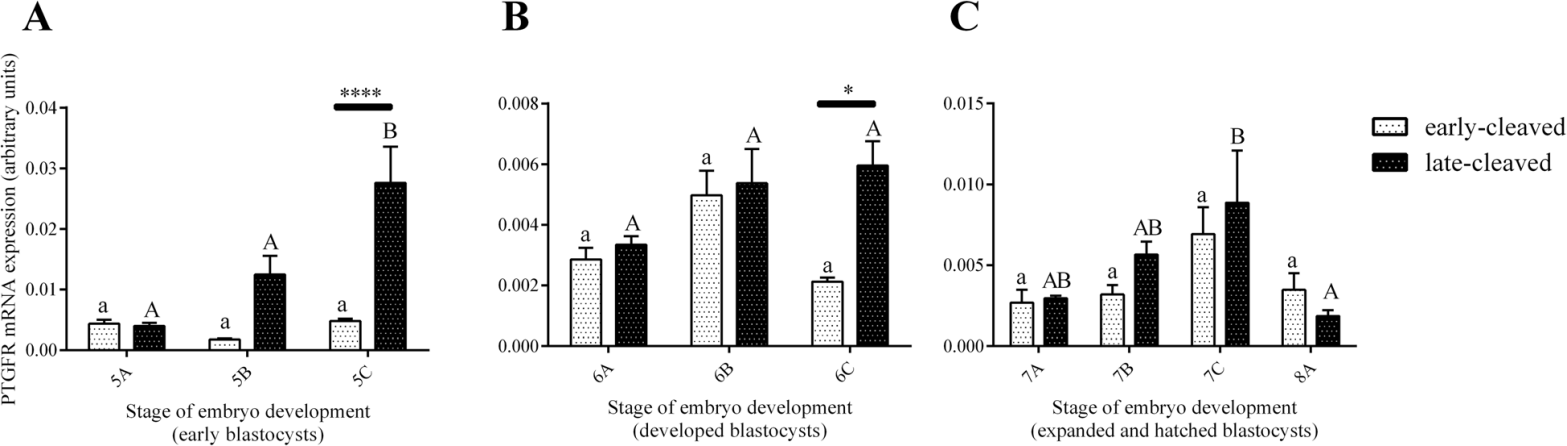
The transcription profile of PTGFR in bovine early- and late-cleaved embryos at the blastocyst stage. This figure demonstrates the changes in mRNA expression in (**a**) early blastocysts (5), (**b**) blastocysts (6) at grades A, B and C, and (**c**) expanded blastocysts (7) at grades A, B and C and grade A hatched blastocysts (8) within each group. The values are presented as arbitrary units and are expressed as the mean ± SEM. Different small letters indicate statistical significance (P < 0.05) within the early-cleaved group (white bars), different capital letters indicate significant differences (P < 0.05) within the late-cleaved group (black bars), and asterisks indicate statistical significance (P < 0.05) between the early- and late-cleaved groups, as determined by two-way ANOVA, followed by the Bonferroni’s multiple comparison test

Figure 3 shows the PGFS mRNA expression in the 2-, 4-, 8-, and 16-cell stages of the early- and late-cleaved embryo groups. In the early-cleaved group, we did not find any differences in PGFS expression between the 2-, 4- or 8-cell embryos, except from its significant decrease at the 16-cell stage compared to the 8-cell stage (*P* < 0.05). We also found that within late-cleaved group the PGFS mRNA expression was relatively stable between the 2-, 4- and 16-cell stages, but it was significantly higher at 16-cell stage compared to the 8-cell stage of embryo development. (P < 0.05). Comparing the PGFS mRNA expression between the early- and late-cleaved groups, we observed that it was significantly higher in the late-cleaved group at the 2-, 4- and 16-cell stages (*P* < 0.05). Figure 4 shows the PGFS transcript abundance in different quality morulas. We did not find any significant differences between all the analysed groups of the obtained morulas (*P* > 0.05). Figure 5 presents the PGFS mRNA expression in the following blastocyst stages: early blastocysts (stage 5), blastocysts (stage 6), expanded blastocysts (stage 7) and hatched blastocysts (stage 8). Within the late-cleaved group of early blastocyst, we found a significantly higher PGFS mRNA expression in the embryos grade A than in the grade C (*P* < 0.05). Moreover, we observed that in grade A early blastocysts, the expression of the PGFS transcript was significantly higher in the late-cleaved group than in the early-cleaved group (*P* < 0.05). Within the group of developed blastocysts, we showed significantly higher PGFS mRNA expression levels in grade B blastocysts than in grade C blastocysts in the early-cleaved group (P < 0.05). In grade C blastocysts of stage 6, we observed significantly higher PGFS transcript abundance in blastocysts from the late-cleaved group than in the early-cleaved group (P < 0.05). We found that within the early-cleaved group of expanded blastocysts, the PGFS mRNA expression increased from grade A to grade C. Additionally, in the early-cleaved group, we observed significantly higher mRNA expression in grade A hatched and grade C expanded blastocysts than in grade A and B expanded blastocysts (*P* < 0.05). Within the late-cleaved group of expanded and hatched blastocysts, we observed higher PGFS mRNA expression levels in hatched grade A blastocysts than in expanded grade A blastocysts but not in grades B and C. Additionally, we showed that there was significantly higher levels of PGFS mRNA expression in the late-cleaved group than in the early-cleaved group of the hatched blastocysts (*P* < 0.05). Figure 6 shows the PTGFR mRNA expression in the 2- to 16-cell embryos from the early- and late-cleaved groups. Within the late-cleaved group, we observed that the PTGFR mRNA abundance was significantly higher in embryos at the 2- and 16-cell stages than in the 4- and 8- cell stages (*P* < 0.05). Moreover, we observed that the PTGFR mRNA expression was significantly higher in the 2- and 16-cell embryos in the late-cleaved group than in the early-cleaved group (P < 0.05). Figure 7 shows the PTGFR mRNA expression at the morula stage. We observed that within the late-cleaved group, the PTGFR transcript abundance was significantly higher in grade C morulas than in grade A and B morulas (*P* < 0.05). Moreover, in the grade C morulas, the PTGFR mRNA expression was significantly higher in the late-cleaved group than in the early-cleaved group (*P* < 0.05). Figure 8 presents the PTGFR mRNA expression in the early developed blastocysts as well as in the expanded and hatched blastocysts. We found that in the early blastocysts, the PTGFR mRNA expression increased from type A to type C in the late-cleaved group only. In this group, the PTGFR transcript abundance was significantly higher in the early blastocysts of grade C than those in grades A and B (*P* < 0.05). Moreover, we observed significantly higher mRNA expression of this gene in grade C early blastocysts from the late-cleaved group than from the early-cleaved group (P < 0.05). Similarly, we showed significantly higher PTGFR mRNA expression levels in grade C blastocysts from the late-cleaved group than from the early-cleaved group (P < 0.05). Within the expanded blastocysts, we observed an increasing trend of PTGFR mRNA expression from grade A to C in the late-cleaved group, but we did not observe significant differences (*P* > 0.05). We found that in the late-cleaved group, the PTGFR transcript expression was significantly higher in grade C expanded blastocysts than in hatched blastocysts (P < 0.05).

### Immunolocalization of PGFS and PTGFR in bovine preimplantation embryos

**Fig. 9:**
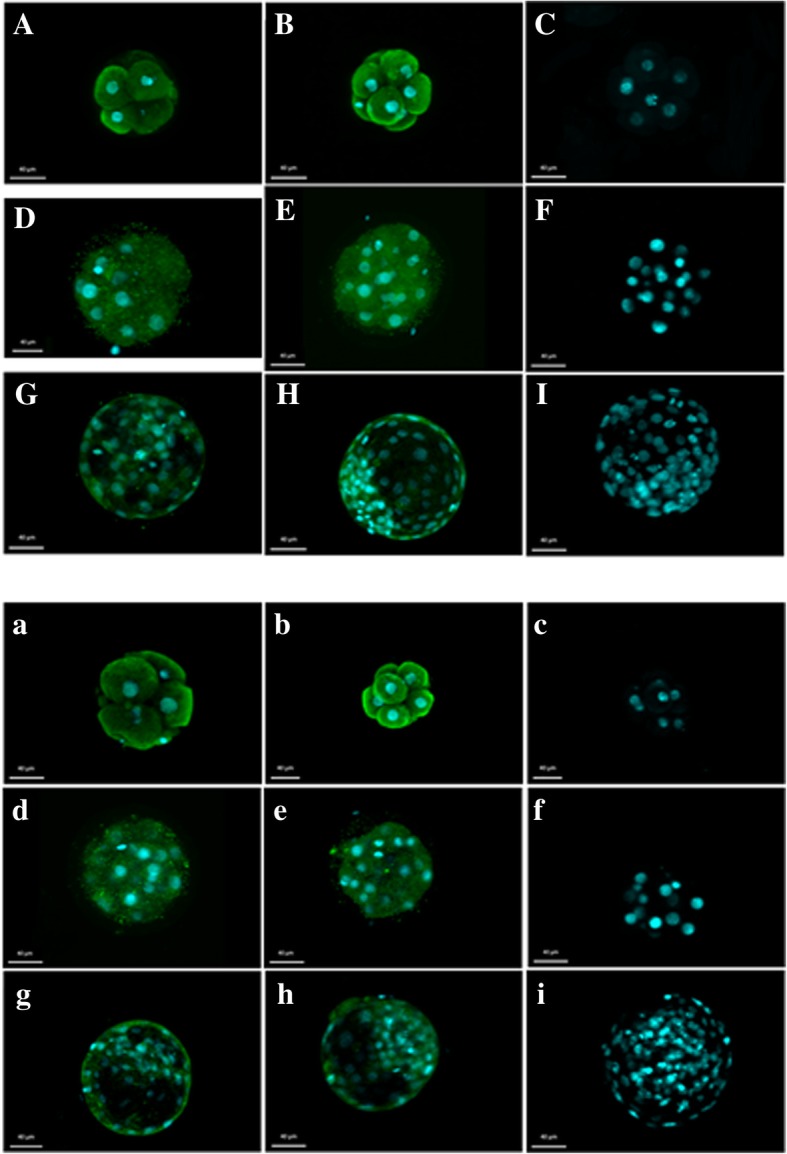
Immunolocalization of PGFS and PTGFR in bovine early- and late-cleaved embryos at early different stages of development. The intracellular localization of PGFS (A, B, D, E, G, H), and PTGFR (a, b, d, e, g, h) was detected by green fluorescence in the early- and late-cleaved groups of embryos, where A, D, G and a, d, g images represent the early-cleaved embryos, but the B, E, H and b, e, h images represent the late-cleaved groups of embryos. The nuclei of the cells are depicted by blue fluorescent dots. Panels C, F, I and c, f, I present images of the negative controls for PGFS and PTGFR, respectively. A-C - images of PGFS staining in the early stages of development. a-c – images of PTGFR staining in the early stages of development. D-F - images of PGFS staining in the morula stage of embryo development. d-f - images of PTGFR staining in the morula stage of embryo development. G-I - images of PGFS staining in the blastocyst stage of embryo development. g-i - images of PTGFR staining in the blastocyst stage of embryo development

Figure 9 presents the immunolocalization of proteins for PGF_**2α**_ synthase and its receptor in 2- and 16-cell embryos, morulas and blastocysts from the early- and late-cleaved groups. We confirmed the presence of PGFS and PTGFR in all analysed embryos. The intracellular region was depicted by green immunofluorescence, but the nuclei were located with blue dye. Negative control experiments for the immunofluorescence localization of PGFS and PTGFR were also performed.

### The production of PGF_2α_ by bovine preimplantation embryos

**Fig. 10:**
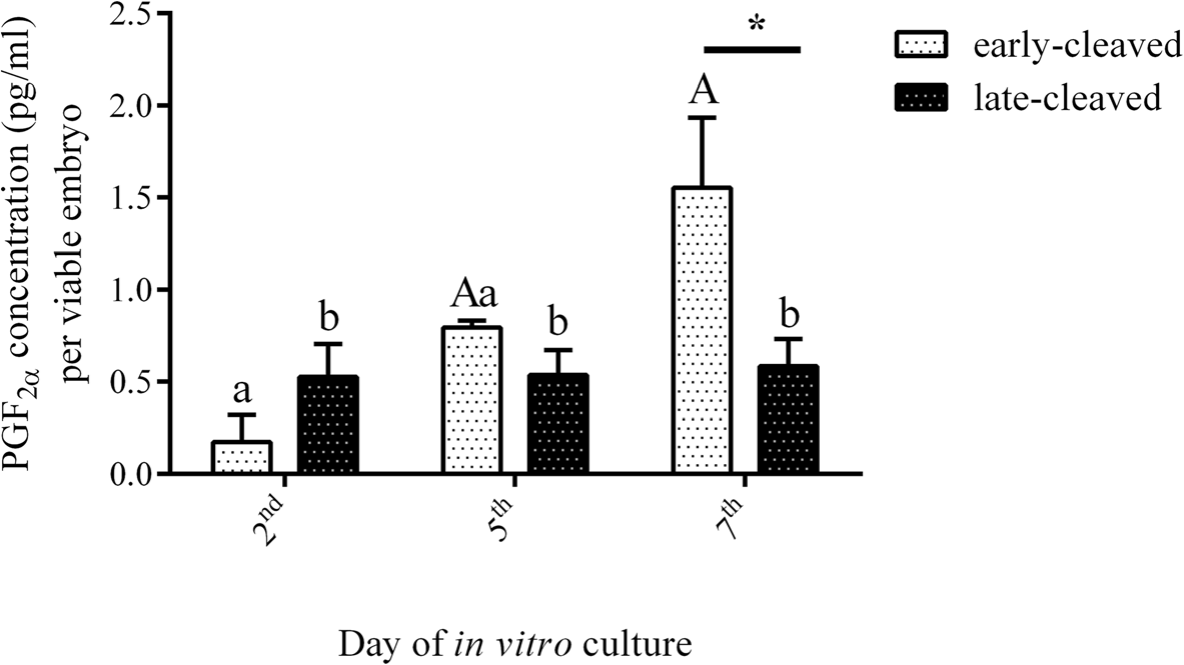
Prostaglandin F_2α_ concentrations in the culture medium of day 2, day 5 and day 7 bovine early- and late-cleaved embryos. The levels of PGF_2α_ are presented as ng/ml per viable embryo and are expressed as the mean ± SEM. Different small letters indicate statistical significance (*P* < 0.05) within the early-cleaved group (white bars), different capital letters indicate significant differences (*P* < 0.05) within the late-cleaved group (black bars), and asterisks indicate statistical significance (*P* < 0.05) between the early- and late-cleaved groups, as determined by two-way ANOVA, followed by the Bonferroni’s multiple comparison test

Figure 10 shows the PGF_**2α**_ concentration in the culture medium from days 2, 5 and 7 of the in vitro culture. The results showed that in the early-cleaved group, the concentration of PGF_**2α**_ increased from day 2 to day 7 of in vitro culture. Within the early-cleaved group, the content of PGF_**2α**_ in the in vitro culture medium was significantly higher on day 7 than on day 2 (*P* < 0.05). Among the late-cleaved group, there were no differences in the PGF_**2α**_ concentration. Nonetheless, we observed significant differences in the PGF_**2α**_ concentration in the day 7 in vitro culture medium between the early- and late-cleaved groups (P < 0.05).

## Discussion

It is well known that embryos produced in vivo are of a higher quality in relation to their counterparts produced in vitro [19, 20]. Moreover, until the blastocyst stage, embryos have some autonomy. The oocytes aspirated from the ovarian follicles can be inseminated in vitro and cultured for approximately 7 days. During this time, they do not need exposure to the maternal reproductive tract to establish pregnancy. Similarly, the recipient’s oviduct does not need contact with the embryo to establish pregnancy during the embryo transfer procedure [20]. Therefore, a detailed examination of the molecular mechanisms regulating preimplantation embryo development could increase the basic knowledge about the in vitro embryo production procedure and could identify factors involved in bovine reproduction; this will improve the procedure itself and will help to optimize reproductive efficiency for livestock production. To accomplish these goals, we should understand the mechanisms that control preimplantation embryo development and focus on the expression patterns and functions of genes that may be involved during these crucial steps. Prostaglandins are the key mediators of the female reproductive system, including ovulation, fertilization, pregnancy maintenance or parturition [21]. In the present study, we investigated the expression of genes involved in the synthesis and action of PGF_2α_ in two different groups of in vitro cultured bovine embryos at different developmental stages based on the time of their first cleavage. The obtained results proved that in the bovine preimplantation embryos, the mRNA expression of PGFS and PTGFR changed substantially according to the developmental stage of the preimplantation embryo as well as its quality. PGFS mRNA expression has been detected previously in bovine embryos but only at day 7 of in vitro culture [22]. To the best of our knowledge, our report is the first to demonstrate the exact transcript expression of PGFS and PTGFR in bovine embryos from the 2-cell stage to the blastocyst stage. In our research, we observed that in the early stages of embryo development (2-, 4-, and 16-cells), the PGFS mRNA expression was higher in embryos originating from late-cleaved zygotes than from early-cleaved zygotes. This result could indicate that the PGF_2α_ autocrine regulatory pathway exists, which is highly active in late-cleaved embryos. Hatamani et al. [23] demonstrated that during the first embryonic cleavage, major zygotic genome activation occurs. They observed that at the 2-cell and 4-cell stages, important processes are involved in the preparation of basic cellular mechanisms. It was demonstrated that this is the time when embryo development depends on maternal transcription [24]. The literature data show a few models to investigate the oocyte and embryo quality [25]. The model proposed by Lonergan et al. [17] proves that embryos that cleave 48 hpi reflect lower developmental competence than embryos that undergo the first cleavage after 30 hpi. Pers-Kemczyc et al. [26] used this model to investigate chromosome aberrations and the speed of development of early- and late-cleaved in vitro-produced bovine embryos. The authors detected that early-cleaved embryos were less often chromosomally abnormal and underwent divisions with a higher speed than late-cleaved embryos. They also documented that late-cleaved embryos were more often arrested at the 8–16-cell stage, the time of embryo development after which major gene activation (MGA) takes place, than early-cleaved embroys [27, 28]. MGA occurs between the fourth and fifth cell cycles and initializes biological and morphological alterations in preimplantation embryo development [23, 24]. Interestingly, in our study between the 8- and 16-cell stage embryos, we noticed the increased expression of PGFS in the late-cleaved embryos but noticed a decrease in the early-cleaved group. During the early stages of development (2- and 4-cells), the PGFS mRNA expression is under the control of maternal transcription. In the 8-cell stage embryo, when the maternal-to-zygotic genome transition (MZT) occurs, we observed an augmentation of PGFS mRNA expression in the early-cleaved embryos and a decline in the late-cleaved embryos. Finally, in the 16-cell embryo, we documented a decrease in the early-cleaved group and an increase in the late-cleaved group; at this time, the genomic control transitioned to embryonic control. This means that the differences in the PGFS mRNA expression within the early- and late-cleaved groups of embryos depend on the MZT. The next important step during early embryo development is compaction, which is initiated in cows at the 16–32-cell stage and continues until morula formation [4]. During compaction, important processes encompass the cells within cell adhesion molecules and determine trophoblast differentiation and blastocyst morphogenesis [16, 29]. It was documented that incorrect cell-to-cell interactions manifested by the lack of E-cadherin or gap junction disruption may result in a decrease in blastocyst formation, developmental disturbances and embryonic death [30–32]. In our results, we observed that at the time of cavitation, the PGFS mRNA expression was downregulated from the 8- or 16-cell embryos to the morula stage in the early- and late-cleaved embryos, respectively. These changes in the PGFS mRNA expression, which occur during compaction, suggest some relevance of PGF_2α_ synthesis during the time of blastomere polarization and the establishment of the first cell-to-cell contact in embryos, and they lead to the formation of a compact morula. After this developmental step, we did not observe differences in PGFS mRNA expression among all grades of the morula stage up to the blastocyst stage when cavitation takes place. Among the blastocyst stage, we can distinguish early, developed, expanded and hatched blastocysts. In cows, the PGFS mRNA expression was reported by Torres et al. [22] in day 7 pre-hatched blastocysts. Our research shows how the mRNA expression differs between the particular stage and the quality of the blastocysts (according to IETS) obtained from the early- and late-cleaved groups of embryos. At the early blastocyst stage (grade A), we observed that the PGFS mRNA expression was more abundant in the late-cleaved group than in the early-cleaved group. Similarly, we observed high PGFS gene expression in the developed (grade C) and hatched blastocysts obtained from the early- and late-cleaved zygotes. Moreover, we depicted that among the expanded blastocysts from the early-cleaved group, the PGFS mRNA expression was upregulated in grade C blastocysts in relation to their counterparts from grades A and B. Moreover, among the early-cleaved group of blastocysts, the percentage share was the highest for blastocyst 7A but was the lowest for 7C. Our results indicate that the PGFS mRNA expression is commonly high in blastocysts belonging to the late-cleaved group or grade C blastocysts, which are known to reflect low-quality embryos. This suggests that the potential PGF_2α_ autocrine regulatory pathway is more intense in low-quality blastocysts.

In our research, we also analysed the expression pattern of PTGFR in bovine preimplantation embryos. The PGF_2α_ receptor was previously detected in the bovine uterus [33], the corpus luteum [34] and blastocysts [35]. The PGF_2α_ receptor belongs to the G-protein coupled receptors whose activation stimulates the release of the intracellular Ca^2+^ concentration, which consequently activates protein kinase C (PKC) [36]. Gao et al. [37] demonstrated that in the bovine endometrium, PCK-dependent PTGFR activation stimulated the expression of factors involved in cell proliferation, migration, differentiation, angiogenesis and tissue repair (PTGS-2, VEGF, CTGF, TGFB-1, IL-8). In the porcine endometrium, the increased expression of the PGF_2α_ receptor activated the expression of genes that participate in tissue remodelling and angiogenesis at the time of implantation [38]. It was shown that activation of the PGF_2α_ receptor can result in the disruption of gap and tight junctions, the perturbation of cell-to-cell communication and the induction of apoptosis [39–41]. The above data indicate that the role of PGF_2α_ in important intracellular processes is necessary for proper implantation and cell survival; in addition, these data indicate that PGF_2_ can act in an autocrine and paracrine manner in bovine preimplantation embryo development.

We detected that PTGFR expression was significantly higher in the late-cleaved group of 2- and 16-cell stage embryos than in the early-cleaved group. Moreover, among the late-cleaved group, we detected the downregulation of PTGFR transcript abundance between the 2- and 4-cell stages; we also detected upregulation between the 8-cell and 16-cell stages of development. These results suggest that after embryonic genome activation, in the late-cleaved group, these changes could be related to major zygotic genome activation and MGA processes, as mentioned above. Analysis of the morula stage of development showed that the PTGFR mRNA expression in the late-cleaved group was significantly higher in grade C morulas than in grade A and B morulas. Within grade C morulas the transcript abundance for PTGFR was higher in the late-cleaved group than in the early-cleaved group. Additional analysis of PTGFR expression between the 2- and 16-cell embryos and the morula stage indicated that in late-cleaved embryos, the mRNA transcript abundance was significantly higher in grade C morula compared to that in all remaining stages. This suggests that in low-quality embryos, the processes that take place during MGA and compaction can be regulated by PGF_2α_ in a paracrine manner. Analysis of PTGFR expression among the blastocyst stage showed the stable expression of this gene in the early-cleaved group and the upregulation from grade A to grade C among the early and expanded blastocysts belonging to the late-cleaved group of embryos. Furthermore, during the analysis of the early and developed grade C embryos, which reflect a low quality, we observed a high expression of PTGFR in the late-cleaved group of embryos. Our results suggest that the low quality of embryos reflects a high level of the PTGFR transcript. In the literature, we found a widely described negative influence of PGF_2α_ on embryo quality and development [10, 11, 15]. Kim et al. [42] observed that PGF_2α_ addition to the culture medium decreased the mRNA expression of PTGS2, the correct level of which is correlated with proper blastocyst implantation, trophoblast proliferation and embryo-maternal interaction. They suggested that the downregulation of PTGS2 synthesis can reduce the embryo quality. This research group indicated that PGF_2α_ suppressed SSLP1 expression, for which the increased expression was positively correlated with blastocyst hatching. Moreover, they described that CASP3, which is considered one of the main apoptotic factors, was upregulated in bovine blastocysts after stimulation of PGF_2α_. According to the above data (which show that the addition of PGF_2α_ to the culture medium decreased the number of blastocysts and pregnancies) as well as our results (which indicate a high PTGFR expression in low-quality embryos), we can assume that the level of PTGFR expression in the bovine embryos can reflect their developmental competences. The trend of high PTGFR transcript abundance in low-quality embryos in connection with the high PGF_2α_ synthesis possibility (expressed by PGFS mRNA expression) indicate that in vitro-produced bovine embryos might be the target and the source of PGF_2α_.

The measurement of the PGF_2α_ concentration in the culture medium at days 2, 5 and 7 showed that the examined bovine preimplantation embryos from both the early- and late-cleaved groups were able to produce PGF_2α_ in different patterns. Our results showed that PGF_2α_ production changes during in vitro culture and depends on the quality of the embryos. PGF_2α_ produced by in vitro cultured embryos has been reported previously in rabbits [43] sheep [44], mares [45], sows [46], women [47] and cows [35, 48]. The expression of the receptor for PGF_2α_ was reported in the bovine endometrium and myometrium [34]. Different studies indicate that PGF_2α_ secretion as well as PGFS and PTGFR expression are positively correlated with endometrium growth during the oestrous cycle and early pregnancy [49]. Kaczyński et al. [38] demonstrated that PGF_2α_ can regulate the expression of genes involved in the establishment of pregnancy and angiogenesis in the endometrium during early pregnancy in sows. In the ovine uterus, PGF_2α_ upregulated ANGPTL3 gene expression, which is associated with angiogenesis and consequently increased uterine blood flow during early pregnancy [50]. Zhang et al. [49] demonstrated the influence of PTGFR agonists on cell proliferation and growth factors in the bovine endometrium during the oestrous cycle and early pregnancy. Moreover, activation of the PGF_2α_ receptor may take part in the regulation of genes involved in endometrial repair during the oestrous cycle in the cow [37]. Our results showed that PGF_2α_ production in the culture medium increased from day 2 to day 7 of in vitro culture in the early-cleaved group of embryos and was stable in the late-cleaved group of embryos. The obtained data prove that PGF_2α_ production is high when the embryo reaches the blastocyst stage and implantation occurs. This is consistent with the observation that PGF_2α_ can impose a paracrine effect on the endometrial tissues and, in this way, may influence the processes involved in implantation, uterine receptivity and the establishment of pregnancy [22]. Moreover, we observed significantly higher concentrations of PGF_2α_ in the day 7 culture medium obtained from the early-cleaved group than those from the late-cleaved group. Pers-Kamczyc et al. [26] noticed that early-cleaved embryos reflect high quality. These results suggest that PGF_2α_ production increased during in vitro culture in the early-cleaved group of embryos. PGF_2α_ is produced more abundantly at day 7 of IVP by the early-cleaved group than by the late-cleaved group. The above data suggest that this prostanoid could reflect the quality of bovine embryos produced in vitro.

## Conclusions

In summary, we described the mRNA expression of genes involved in PGF_2α_ synthesis and the development of bovine preimplantation embryos from the 2-cell to the hatched blastocyst stage obtained from early- and late-cleaved zygotes. Additionally, the presence of PGFS and PTGFR in the analysed embryos was confirmed by immunofluorescence staining. The mRNA expression of PGF_2α_ synthase and its receptor depends on the developmental stage and embryo quality. Moreover, analyses of PGFS and PTGFR expression in bovine blastocysts and of PGF_2α_ embryo production suggest that prostaglandin F_2α_ can act in an auto- and paracrine manner in bovine in vitro-produced preimplantation embryos. Our results show the tendency of PTGFR and PGFS mRNA expression to be upregulated in embryos with low developmental potential, which can indicate that some compensation mechanism related to high PGFS and PTGFR mRNA expression exists among late-cleaved, low-quality embryos. Moreover, late-cleaved embryos reflect high cleavage rates, and during in vitro culture, they develop to the blastocyst stage, which can indicate the possibility of using them in ART techniques just as early-cleaved embryos are used. However, the usefulness of these embryos and the implantation and pregnancy rates need to be confirmed with future research. Our data confirmed the utility of the model for examining the quality of embryos based on the time of first cleavage. The dynamic changes in mRNA expression of PGF_2α_ synthase and its receptor in the analysed group of embryos could indicate that these factors can alter the embryonic development of in vitro-produced bovine preimplantation embryos.

## Methods

### Cumulus-oocyte complex collection

The described animal procedures were carried out after providing the approval from the Local Animal Care and Use Committee in Olsztyn, Poland (Agreement No. 76/2014/DTN). The material for all experiments were the ovaries obtained from non-pregnant Holstein cows with normal cycles from a local slaughterhouse (“Warmia”, Biskupiec, Poland). The ovaries were collected in the vial containing sterile 0,9% saline solution at 37 °C within 20 min of the slaughtered and were subjected to further processing in the laboratory after 40 min.

Aspiration of follicular fluid from less than 5 mm diameter subordinate ovarian follicles gave the cumulus-oocyte complexes (COCs). The oocytes were separated under the assessment by stereomicroscope and only COCs surrounded by cumulous cells homogenously by at least three layers of cumulus cells and without signs of ooplasm degradation were taken for the future studies. The COCs were washer two times in wash medium (M199; #M5017, Sigma Aldrich, Germany) supplemented with 20 mM HEPES (#H4034 Sigma Aldrich, Germany), 25 mM sodium bicarbonate (#S4019 Sigma Aldrich, Germany), 0,4% bovine serum albumin (#A9418 BSA; Sigma Aldrich, Germany) and 40 μg/ml gentamicin (#G1272, Sigma, Aldrich, Germany) and subsequently one time in maturation medium.

### In vitro embryo production

After segregation, groups of 50 COCs were placed in four-well plates (#144444, Thermo Fisher Scientific, USA) containing 400 μl of maturation medium (TCM 199 Maturation Medium (19,990/0010, Minitube, Germany) suplemented with 0,02 IU/ml pregnant mare’s serum gonadotropin (PMSG, #G4527, Sigma Aldrich, Germany), 0,01 IU/ml human chorionic gonadotropin (hCG, #C0684, Sigma Aldrich, Germany) and 5% foetal bovine serum (FBS, #12106C, Sigma Aldrich, Germany) overlaid with 400 μl of culture oil (#M5310, Sigma Aldrich, Germany). The maturation was conducted for 24 h in humidified air atmosphere at 38.5 °C in 5% CO_2_. Thereafter, COCs were washed and fertilized in vitro in fertilization medium (TL fertilization medium 19,990/0030, Minitube, Germany), containing 10 μg/ml heparin (#H3393, Sigma Aldrich, Germany), 20 mM sodium pyruvate (#P3662, Sigma Aldrich, Germany) and 0,6% BSA using frozen semen obtained from the same bull. The semen was thawed and capacitated through the swim-up procedure in capacitation medium (TL sperm capacitation medium, 19,990/0020, Minitube, Germany) supplemented with 1 mM sodium pyruvate, 0,6% BSA and 0,1 mg/ml gentamicin for 1 h under the same conditions (38.5 °C, 5% CO_2_ humidified air atmosphere). After recovering the motile sperm, which was located in the upper two-thirds of the medium, it was centrifuged at 200×g for 10 min. For the fertilization only the sperm was used, which diluted in fertilization medium. The fertilization was conducted by co-incubation of 25 COCs groups with semen (concentration 10^6^/ml) in four-well dishes containing 400 μl of fertilization medium overlied by 400 μl of mineral oil. The day of semen capacitation and in vitro insemination was regarded as day 0. After 30 h of incubation at 38.5 °C in a 5% CO_2_ humidified air atmosphere, the embryos were secluded from the cumulus cells by vortexing and followed by washing three times in the wash medium. The early- and late-cleaving groups of embryos were separated according to Patel et al. [2007]. The embryos cleaved 30 h after fertilization provided the group of early-cleaving embryos (*n* = 5; each pool consisted of five embryos for RNA extraction). The group of the late-cleaving embryos (n = 5; each pool consisted of five embryos for RNA extraction) were obtained from the same wells 36 h after fertilization. The early- and late-cleaving embryos were cultured in groups of 50 embryos in four-well dishes containing 400 μl culture medium (SOF; synthetic oviduct fluid medium; 19,990/0040, Minitube, Germany) supplemented with amino acids (10 μl/ml BME (#B6766, Sigma Aldrich, Germany) and 20 μl/ml MEM (#M7145, Sigma Aldrich, Germany), 3,3 mM sodium pyruvate and 5% FBS overlied by 400 μl of mineral oil. The embryos were incubated at 38.5 °C in an atmosphere of 5% CO_2_, 5% O_2_, and 95% N_2_ with high humidity up to day 8 of in vitro culture.

The developmental rates and quality for embryos from the early- and late-cleaving zygotes were provided by morphological examination on a stereo- or inverted microscope at 20, 50 or 100x magnification according to the International Embryo Transfer Society (IETS).

### Sample collection for RNA isolation and reverse transcription

The pools of 5 embryos from early- and late-cleaving group at different stages of in vitro development suspended in the extraction buffer were used for RNA isolation according to the manufacturer’s instructions (#KIT0204, Arcturus PicoPure RNA Isolation Kit, Applied Biosystems, USA). For remove of genomic DNA contamination the RNase-Free DNase Set was used (#79254, Qiagen, Germany). After isolation, the samples were stored at − 80 °C. Reverse transcription (RT) was assessed using oligo (dT)12–18 primers (#18418–012, Invitrogen, USA) by Super Script III reverse transcriptase (#18080–044, Invitrogen, USA). The total volume of RT reaction stand at 20 μl. The reverse transcription reactions were performed by incubation at 65 °C for 5 min and 42 °C for 60 min, followed by 15 min step of denaturation at 70 °C. For degradation of the RNA strand of RNA-DNA hybrids we conducted by adding RNase H (#18021–071, Invitrogen, USA) to the samples (37 °C for 20 min). After RT, obtained products were stored at − 20 °C until real-time PCR amplification.

### Quantitative real-time PCR

The expression of mRNA for PGFS, PTGFR, and GAPDH was measured by real-time PCR using specific primers and was performed with an ABI Prism 7900 Sequence Detection System (Applied Biosystems, Life Technologies, USA). For the normalization of obtained results of mRNA expression were used the glyceraldehyde-3-phosphate dehydrogenase (GAPDH) as an internal control and the mRNA abundance was expressed as arbitrary units. Three candidate genes: GAPDH, β-actin and H2A.1 were compared by NormFinder software for choosing the housekeeping gene [51]. Designing of primers was conducted by online software package (http://bioinfo.ut.ee/primer3/) and chosen sequences and the sizes of the amplified fragments of all transcripts are presented in Table 3. The Real-time PCR procedure was performed using Maxima™ Probe/ROX qPCR Master Mix (2X) (# K0222, Thermo Scientific, USA) in 384-well plates contained 3 μl of RT product (quantity of cDNA equivalent to that from 0.15 blastocysts), 1 μM each of the forward and reverse primers and 5 μl SYBR Green PCR Master Mix per each well. Real-time PCR was conducted in following steps: 95 °C for 10 min, followed by 40 cycles of 94 °C for 15 s and 60 °C for 60 s. We used the blank samples (only buffer or in the absence of the reverse transcriptase enzyme) to eliminate the possibility of genomic DNA contamination in the RNA samples. After Real-time PCR, we confirmed the specificity of the PCR products by gel electrophoresis and sequencing. The relative quantification of the mRNA expression levels was measured by real-time PCR Miner algorithm [52].

**Table 3:** Primers used for Real-time PCR

Gene symbol	GenBank accession No.	Primer sequence 5′ to 3′	Amplicon size (bp)
GAPDH	NM_001034034.2	CACCCTCAAGATTGTCAGCA GGTCATAAGTCCCTCCACGA	103
PGFS	S54973.1	GGAGGACCCCAGGATCAAAG CTCAGCAATGCGTTCAGGTG	130
PGFR	D17395	TCAGCCCTCACCCAGATAGT GGCCATTTCACTGTTCAGG	247

### Immunofluorescence

The immunofluorescence staining was conducted for selected embryos. At the first step of staining the embryos were fixed in 4% paraformaldehyde for 1 h at 4 °C, washed in PBS, and permeabilized with 0.5% Triton X-100 for 10 min at room temperature. Permeabilized embryos were washed in PBS and incubated for 1 h in the blocking solution (Novocastra Protein Block, ref. RE7102, Novocastra Laboratories Ltd., Newcastle upon Tyne, United Kingdom). Afterwards, the embryos we incubated with the antibodies diluted 1:200 in blocking solution: rabbit polyclonal anti-PGFS (ab84327, Abcam, United Kingdom) and anti-PTGFR (#101802, Cayman Chemical), USA whose specificity was confirmed previously by WB and IHC. Following overnight incubation (4 °C) embryos were washed in PBS and incubated for 1 h at room temperature with Alexa Fluor 488 donkey anti-rabbit IgG antibody (concentration 1:1000 in blocking solution, A-21206, Invitrogen, Life Technologies, Foster City, CA, USA). For the negative control embryos were incubated only with the secondary antibodies. After final washing in PBS, the embryos were mounted in Vectashield with DAPI (#H-1200, Vector Laboratories, USA). The analysis of protein immunostaining was conducted under an Olympus Fluoview FV10i confocal laser scanning microscope.

### ELISA assay

The PGF_2α_ concentration in the culture medium was determined. The samples were collected on days 2, 5 and 7 post-insemination with the time of first cleavage taken into account. The concentration of PGF_2α_ in the conditioned media was measured using the PGF_2α_ high sensitivity ELISA kit (#ADI-931-069, Enzo Life Sciences, USA) according to the manufacturer’s instructions. The standard curve for PGF_2α_ ranged from 19.5 to 50,000 pg/ml. The samples were measured in duplicate.

### Statistical analysis

The obtained data were analysed using the statistical software GraphPad PRISM 6.05 (GraphPad Software, Inc., La Jolla, CA, USA). All numerical data are presented as the mean ± SEM. The differences in the mRNA expression and the PGF_2α_ concentration were analysed by two-way ANOVA, followed by Bonferroni’s multiple comparison test. The cleavage and blastocyst rates were analysed by a Chi-square test with Yate’s correction. The differences were considered statistically significant at the 95% confidence level (< 0.05).

## Data Availability

The datasets used and analysed during the current study are available from the corresponding author on reasonable request.

## Abbreviations

ANGPTL3: Angiopoietin like 3
ANOVA: Analysis of variance
ART: Assisted reproductive technology
BSA: Bovine serum albumin
CASP3: Caspase 3
cDNA: Complementary DNA
DAPI: 4′,6-diamidino-2-phenylindole
ELISA: Enzyme-linked immunosorbent assay
hpi: hours post-insemination
mRNA: messenger RNA
PCR: Polymerase chain reaction
SSLP1: Secreted seminal-vesicle Ly-6 protein 1
